# Metabolic reprogramming of T regulatory cells in the hypoxic tumor microenvironment

**DOI:** 10.1007/s00262-020-02842-y

**Published:** 2021-02-03

**Authors:** Varun Sasidharan Nair, Reem Saleh, Salman M. Toor, Farhan S. Cyprian, Eyad Elkord

**Affiliations:** 1 Cancer Research Center, Qatar Biomedical Research Institute (QBRI), Hamad Bin Khalifa University (HBKU), Qatar Foundation (QF), Doha, Qatar; 2 Department of Basic Medical Sciences, College of Medicine, Member of QU Health, Qatar University, Doha, Qatar; 3 Biomedical Research Center, School of Science, Engineering and Environment, University of Salford, Manchester, M5 4WT UK

**Keywords:** T regulatory cells, Hypoxia, Tumor microenvironment, Metabolism, Glycolysis, Fatty acid metabolism

## Abstract

Metabolic dysregulation in the hypoxic tumor microenvironment (TME) is considered as a hallmark of solid tumors, leading to changes in biosynthetic pathways favoring onset, survival and proliferation of malignant cells. Within the TME, hypoxic milieu favors metabolic reprogramming of tumor cells, which subsequently affects biological properties of tumor-infiltrating immune cells. T regulatory cells (Tregs), including both circulating and tissue-resident cells, are particularly susceptible to hypoxic metabolic signaling that can reprogram their biological and physicochemical properties. Furthermore, metabolic reprogramming modifies Tregs to utilize alternative substrates and undergo a plethora of metabolic events to meet their energy demands. Major impact of this metabolic reprogramming can result in differentiation, survival, excessive secretion of immunosuppressive cytokines and proliferation of Tregs within the TME, which in turn dampen anti-tumor immune responses. Studies on fine-tuning of Treg metabolism are challenging due to heterogenicity of tissue-resident Tregs and their dynamic functions. In this review, we highlight tumor intrinsic and extrinsic factors, which can influence Treg metabolism in the hypoxic TME. Moreover, we focus on metabolic reprogramming of Tregs that could unveil potential regulatory networks favoring tumorigenesis/progression, and provide novel insights, including inhibitors against acetyl-coA carboxylase 1 and transforming growth factor beta into targeting Treg metabolism for therapeutic benefits.

## Introduction

Cancers are polygenic diseases initiated by multiple oncogenic factors that dysregulate the expression of tumor suppressor genes and/or proto-oncogenes leading to malignant progression [1]. The neoplastic tissue is comprised of heterogeneous population of tumor cells, in a milieu of immune (e.g., myeloid cells, lymphocytes, and natural-killer cells), and non-immune cells (e.g., fibroblasts and endothelial cells) embedded in the extracellular matrix with a plethora of cytokines and chemokines, known as tumor microenvironment (TME) [2–4]. TME has dynamic attributes with pro- and anti-tumorigenic properties, which can also influence drug responses [5]. Tumor cells evade host-immunosurveillance by recruiting surplus of immunosuppressive cells including T regulatory cells (Tregs) [6, 7] and myeloid-derived suppressive cells (MDSCs) [6], which suppress the proliferation of cytotoxic T cells (CTLs) and favor malignant progression [8]. Amongst these suppressive cells, Tregs are considered as the master-regulatory cells, which not only secrete cytokines that promote onset and proliferation of malignancies, but also play indispensable roles in the induction of neo-angiogenesis and metastasis [9–12]. Accumulating evidence suggest that Treg infiltration was evident in vast majority of solid tumors including breast [7], colon [6], pancreatic [13] and ovarian cancer [9]. Tumors samples from advanced stages of cancer exhibit higher infiltration of Tregs, compared with samples obtained from early stages of cancer [14]. Moreover, meta-data analyses showed that higher Treg infiltration is negatively correlated with cytotoxic CD8^+^ T cell infiltration and that is associated with poor-disease prognosis [15]. Currently, it is believed that Treg infiltration favors tumor progression and dampens anti-tumor immune responses; thus, it is essential to understand the progression and functions of Tregs in the TME [16, 17].

Tumor cells adapt to multiple metabolic processes including glycolysis, oxidative phosphorylation (OXPHOS) and fatty acid metabolism to obtain energy for their survival and progression in adverse tumor milieu [18]. Moreover, the differentiation of T cells within the TME is indirectly regulated by tumor-mediated metabolites and favors tumor progression [19]. Within the TME, metabolic reprogramming of T cells is initiated by the activation of T cell receptor (TCR) signaling along with various costimulatory molecules, resulting in the production of sufficient ATP to meet energy requirements for T cell proliferation and effector functions [20]. Interestingly, T cells isolated from the TME frequently exhibit exhaustive T cell markers and possess distinct metabolic signatures including reduction in the uptake of glucose and upregulation of reactive oxygen species (ROS) [21]. These metabolic defects could be circumvented and partially restored the activation of tumor-infiltrating CD8^+^ T cells (TILs) through the adequate supplementation of pyruvate and neutralization of ROS [21]. These reports suggest that tumor metabolic environment could alter the regulation, function and tumor-antigen recognition of T cells, leading to inadequate anti-tumor responses.

It has been reported that accumulation of lactate and carbon dioxide could efficiently reprogram the metabolic potentials of tumor cells, including elevated nutrient uptake and glucose metabolism and favor the differentiation of Tregs by inhibiting the infiltration of effector T cells within the TME [22, 23]. Moreover, hypoxic conditions as a result of increased tumor growth and oxygen deprivation stabilize the expression of hypoxia‐inducible factor 1‐α (HIF1‐α), which in turn mediates the induction of FoxP3 expression and favors Treg stability [24, 25]. Therefore, comprehensive analyses of malignancy-induced metabolic/hypoxic regulation of T cells can improve current immunotherapeutic modalities. Numerous studies have focused on the metabolic reprogramming of tumor cells and their influence over T cell function within the TME; however, limited data are available on the metabolic-induced alterations in Tregs in the TME. This review highlights the metabolic reprogramming of physicochemical characteristics of Tregs, their function, differentiation and crosstalk within the TME. Additionally, we focus on the potential metabolic pathways of Tregs within the TME, which may be targeted for improvement of prognosis and development of novel therapeutic strategies.

## Metabolism in the tumor microenvironment

Tumor cells are characterized by their competence to adapt with altering environmental cues by exploiting various nutrients to uphold their necessitating anabolic requirements [3]. This sustained energy demand is accomplished by adequate supply of nutrients and oxygen via tumor vasculature [26]. Consequently, these extracellular nutrients are indispensable for cancer cells to meet their high-energy demand during rapid, uncontrolled proliferation [26]. Unlike normal cells, malignant cells have higher metabolic plasticity, which could reshape the environment even in nutrient-deprived conditions per se [27]. This plasticity has profound influence on tumor differentiation and gene expression within the TME [27]. In this context, Pavlova and colleagues classified tumor-associated metabolic modifications into six groups: (1) deregulation in glucose and amino acid metabolism, (2) altered nutrient uptake, (3) utilization of intermediates from citric acid cycle (TCA cycle)/glycolysis for the biosynthesis of nicotinamide adenine dinucleotide phosphate (NADPH), (4) increased nitrogen requirement, (5) variations in the regulation of metabolite-dependent gene expression and (6) interactions between metabolic pathways within the TME [27].

It has been reported that the highly proliferating cancer cells modify the metabolic components of the TME. For instance, malignant cells take up higher amount of glucose leading to the biosynthesis of large amount of lactate, which could influence many cell populations within the TME [28]. Higher accumulation of lactate creates an immune-subversive milieu by reducing dendritic and T cell activation and migration of tumor-associated macrophages/monocytes [28, 29]. Moreover, the excess accumulation of lactate polarizes resident macrophages to highly activated/ immunosuppressive M2 state and promotes angiogenesis [30, 31]. Excess levels of lactate also favor the biosynthesis of hyaluronic acid by fibroblasts, contributing to higher tumor invasiveness [32].

Hypoxia-inducible factor 1-alpha (HIF-1α) is the key transcriptional factor of hypoxic cells, a hallmark of the TME, and is a downstream target of glucose transporter-1 (GLUT-1) [33]. During hypoxic conditions, the higher glucose uptake by cancer cells could upregulate the stability of HIF-1α, which in turn leads to the attenuation of anti-tumor immune responses [34]. In HIF-1α-knocked-out murine models, the anti-tumor immune responses of CD8^+^ TILs improve through the activation of peroxisome-activated receptor α (PPARα) signaling and also elevated metabolism of fatty acids [35]. HIF-1α promotes the migration of Tregs in the TME through the upregulation of glycolysis and fatty acid oxidation (FAO) within the TME [36]. Indeed, HIF-1α-deficient Tregs exhibit reduction in glycolytic-driven Treg migration and oxidation of fatty acid-driven immunosuppression, which in turn upregulates anti-tumor immune responses of CD8^+^ TILs [36].

**Fig. 1:**
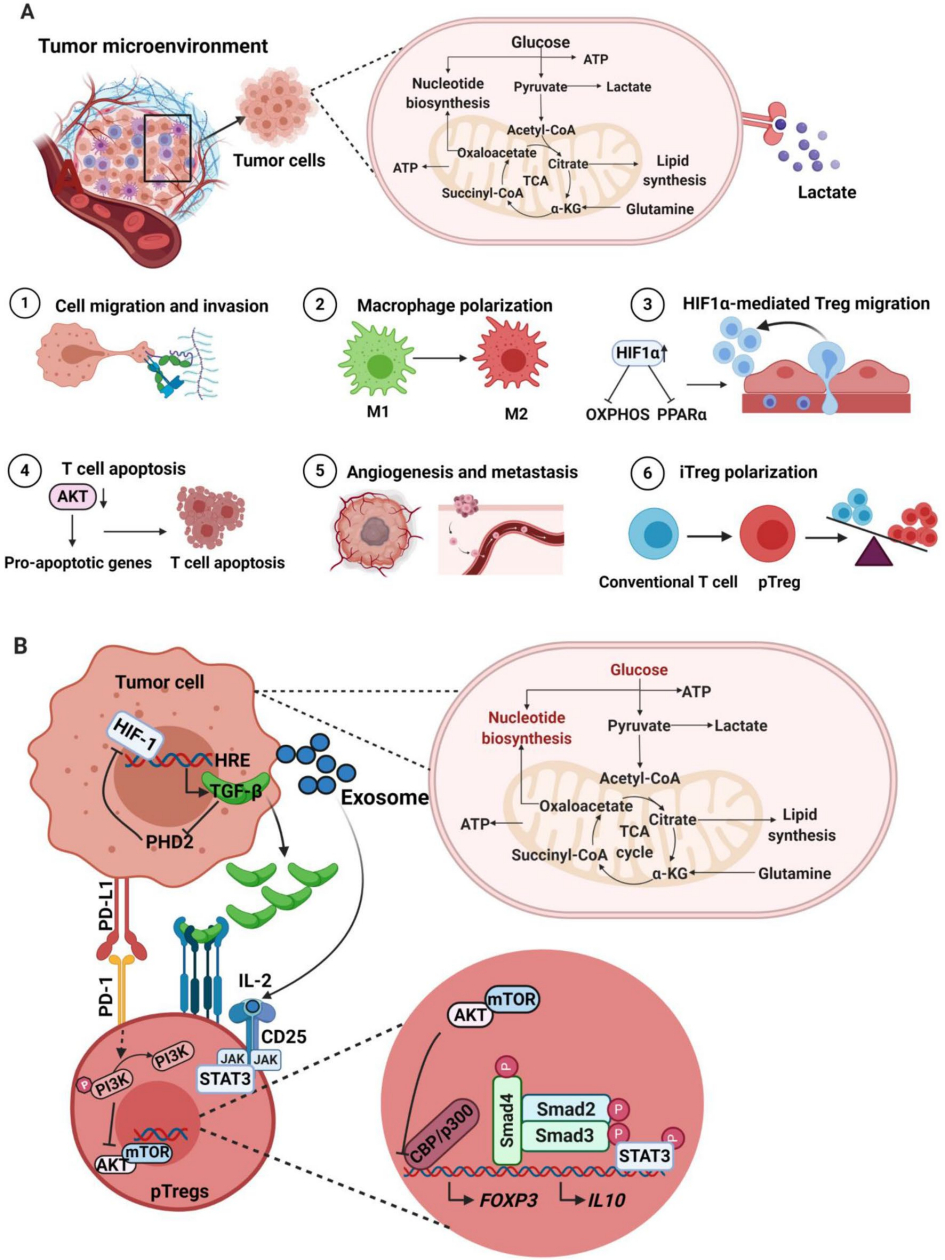
Metabolic effects within the TME (**a**). Tumor cells consume major fraction of glucose and accelerate glycolysis, leading to the accumulation of lactate. The secreted lactate could influence cell types within the TME by activating multiple processes for the survival and proliferation of tumor cells. Higher glycolysis rate within the TME leads to the accumulation of lactate and induces various cellular and molecular events, including the upregulation of hyaluronic acid within the TME and favors tumor migration (**1**); activation of non-immunosuppressive macrophages (M1) to immunosuppressive M2 (**2**); upregulation of HIF-1α on Tregs could inhibit OXPHOS and PPARα signaling and favors the migration of Tregs into the TME (**3**); inhibition of AKT signaling network and induce apoptosis of activated T cells (**4**); promotion of angiogenesis and metastasis (**5**); polarization of conventional T cells to pTregs through the upregulation of TGF-β, HIF-1α, IL-10 and inhibition of AKT/mTOR pathway (**6**). Generation of pTregs within the TME (**b**). In hypoxic TME, HIF-1 binds to the hypoxia inducible response element (HRE) of tumor cells and activates the secretion of TGF-β. The secreted TGF-β also favors HIF-1 expression by inhibiting HIF-1 degradation enzyme PDH2. Moreover, the secreted TGF-β binds to its receptor on pTregs and triggers downstream pathways, including the phosphorylation of SMAD3, binding of phospho-SMAD3 to SMAD4, recruitment of CBP/p300 and binding of these complex on FoxP3 promoter to induce the expression of FoxP3. In addition, PD-L1 is also the downstream target of HIF-1, which could bind to PD-1 on T cells. PD-1/PD-L1 interaction could activate the dephosphorylation of PI3K and block AKT/mTOR pathway. This signaling network helps to stabilize the expression of FOXP3. Moreover, tumor-derived exosomes could activate JAK/STAT3 pathway and favors the upregulation of IL-10. Altogether, hypoxic TME favors the polarization of conventional T cells to pTregs via multiple signaling cascades

Glucose metabolism is the key energy source of T cells for their polarization toward tumor antigen-specific effector T cells. Within the TME, the scarcity of glucose for T cells affects their differentiation to effector T cells. Moreover, a low-glucose milieu could diminish glycolysis of T cells by decreasing serine/threonine-protein kinase (AKT) signaling and inducing the apoptosis of TILs through the upregulation of pro-apoptotic proteins [20]. This metabolic microenvironment may also promote the polarization of naïve CD4^+^ TILs to peripherally induced Tregs [37]. The stimulation of Treg differentiation and their suppressive characteristics is mediated by the metabolic intermediates including kynurenine and tryptophan [38]. In solid tumors, the overexpression of tryptophan-degrading enzymes, including indoleamine-2, 3-dioxygenase (IDO1) and tryptophan-2, 3-dioxygenase (TDO2), catalyzes tryptophan to its derivative, kynurenine [38]. This tryptophan depletion is associated with the apoptosis of effector T cells, within the TME [39]. Additionally, kynurenine promotes the migratory and immunosuppressive characteristics of Tregs through aryl hydrocarbon receptor (AhR)-dependent manner [40]. The overall effect of metabolism in the TME is depicted in Fig. 1a.

## Tregs in the tumor microenvironment

Tregs are key immunosuppressive players, which impede anti-tumor immune responses in the TME [41]. Within the TME, there is cross-talk among Treg and other immune cells, stromal cells, and tumor cells to maintain hypoxic, pro-angiogenic and highly immunosuppressive milieu [4, 8]. Tumor-infiltrating Tregs primarily comprise terminally differentiated and highly suppressive FoxP3high effector Tregs [42]. However, Treg heterogeneity impacts disease outcome across various cancers. Accumulation of FoxP3^+^ Tregs in the TME is concomitant with higher Treg to effector T cell (Teff) ratios, and lower levels of naïve T cells and is associated with worse disease outcomes in various malignancies [43]. However, elevated levels of tumor-infiltrating Tregs have also been associated with good prognosis in certain cancers such as colorectal (CRC) and gastrointestinal cancers [8]. This could be due to Tregs capacity to suppress inflammation in initial stages, associated with disease progression of these cancers. Moreover, this inconsistency can also be attributed to the infiltration of non-suppressive, CD45RA^–^FoxP3^Low^ non-Tregs [44]. Importantly, CRC patients with high levels of effector Tregs exhibited poorer prognosis, while a better prognosis was reported in CRC patients with predominantly higher levels of non-Tregs, who also showed high mRNA levels of TGF-β, TNF-α and IL-12 [45].

Tregs are recruited in the TME by various chemokines including CCR4/8/10 and CCL9/10/11 [8, 46], and expand in the TME in response to various tumor-derived factors [8]. High levels of IL-10, TGF-β and adenosine in the TME promote expansion of natural/thymic Tregs (nTregs) and generation of peripheral Tregs (pTregs) [47, 48]. However, it remains unclear whether Tregs in the TME are trafficked to tumors or are expanded from tissue-resident Tregs [49]. In pancreatic cancer, it has been reported that Tregs are the key source of TGF-β, while their depletion could reprogram fibroblast populations and lead to tumor progression [50]. Moreover, depletion of Tregs leads to upregulation of CCL3, CCL6 and CCL8, and recruitment of immunosuppressive myeloid cells into the TME to favor tumor progression [50]. Furthermore, analyses of TCR repertoire of circulating, tissue-resident and tumor-infiltrating Tregs showed slight overlap with some distinct features in tumor-infiltrating Tregs [51]. Compared with circulation or lymph nodes, tumor-infiltrating FoxP3^high^ effector Tregs are highly activated and express high levels of activation molecules including CD25, ICOS, PD-1, CTLA-4, OX40, GITR and TIGIT [41, 52].

Tumor-infiltrating Tregs suppress proliferation of other effector T cell populations via contact-dependent mechanisms and contact-independent mechanism, primarily through IL-10 and TGF-β secretion [4]. IL-10 and TGF-β secretion suppresses IFNγ and IL-2, which are required for T cell activation and survival within the TME [53, 54]. In addition, secretion of IL-35 by Tregs leads to inhibition of T cell proliferation within the TME [55]. CTLA-4 presents as a homolog of CD28 and is constitutively expressed on Tregs [56]. Interactions between CTLA-4 and CD80/86 on APCs prevent costimulatory signals from CD28 for T cell activation, leading to inhibition of proliferation and cytokine release of effector T cells within the TME [57]. In addition, LAG-3 expression on tumor-infiltrating Tregs and interactions with MHC class II lead to impaired DC maturation and inhibition of proliferation of effector T cell populations [58]. MHC class II interactions are also hampered by increased expression of neuoropilin-1(NRP1) on Tregs, which enhance the suppressive activity and block interactions between APCs and effector T cells via enduring interactions between DCs and Tregs [59, 60]. Lastly, granzyme and perforin expression by tumor resident Tregs induces cytotoxic T cell and NK cell death within the TME in murine models [61].

In addition to immune functions of Tregs in the TME, their potential non-immunologic roles to support tumor progression include supporting angiogenesis, tumor growth/proliferation and transition to metastasis [62–65]. Treg levels in the TME have been correlated with high levels of VEGF [66] and promote angiogenesis by suppression of effector T cells and release of TNFα and IFNγ along with other chemokines such as CXCL9, 10 and 11 [62]. Moreover, murine cancer models have shown that depletion of CD25^+^ Tregs enhanced anti-tumor responses, which correlated with reduction in tumor volume [67]. Other groups have revealed plausible pathways affected by tumor-infiltrating Tregs to support invasion and metastasis in different cancers. For instance, Tan et al. showed that Tregs promote invasion and metastasis in mammary cells via activation of receptor activator of nuclear factor-κB ligand (RANKL) signaling [11], while, Shi et al. showed that tumor-infiltrating Tregs promote invasion of hepatocellular carcinoma via epithelial–mesenchymal transition induced by TGF-β1 secretion [68].

Because of the multi-faceted roles in tumor development and progression across different cancers, tumor-infiltrating Tregs are key targets for multiple therapeutic strategies aimed to counter immunosuppression and promote immune stimulation for clinical benefits.

## Metabolic pathways affecting Treg function in the hypoxic TME

The TME is a dynamic niche orchestrated by heterogeneous metabolic activity of cells with variable vascularity that generate regions of gradient hypoxia. The metabolic features include glycolysis, oxidative stress, OXPHOS, FAO, Warburg effect and amino acid metabolism which can influence the behavior and function of Tregs.

### Treg survival in TME

The metabolic adaptation in TME favor Treg survival in a hostile environment. Recent studies have shown that Tregs exhibit low membrane expression of glucose transporter GLUT-1 along with increased lipid oxidation activity when compared to other effector T cell subsets [37]. Tregs and Teffs exhibit distinct metabolic patterns; the former require glycolysis, and the latter require lipid oxidation for their survival and function [37]. Indeed, Tregs favorably rely on FAO and OXPHOS, especially in low-glucose, high lactate environments with increased NAD/NADH ratios [37]. On the other hand, the pharmacological inhibition of AMPK/lipid metabolism using Etomoxir can reduce in vitro generation of Tregs [37]. This metabolic preference is regulated by the expression of Treg transcription factor FoxP3 that further dampens glycolytic enzymes and Myc expression [69].

### Treg migration into TME

Several metabolic pathways influence Treg migration into the TME. Signaling through CD28 and CTLA-4 ligands has been reported to enhance the migration of Tregs via activation of PI3K-mTORC2 pathway that upregulates glucokinase (GCK) expression, underlying the significance of glycolysis in Treg migratory phenotype in murine models [70]. Furthermore, in vitro migration assays of glioma-produced HIF-1α KO Tregs showed an inhibited migratory response to CCL22 chemotactic agent in GL-261 murine glioblastoma model [71]. On the other hand, high expression levels of amino acid-degrading enzymes including IDO and arginase 1 have been associated with increased Treg infiltration in CRC [72], hepatocellular carcinoma (HCC) [73] and uterine cervical cancer [74]. In concordance with these data, suppression of mTOR decreased IDO1 expression and activity, leading to decreased recruitment of Tregs in the TME in a murine model of medulloblastoma [75].

### Treg expansion in TME

Increased frequency of Tregs in the TME has been associated with a higher proliferation rate of these cells in MCA38 CRC murine model. In this context, signaling via OX40/OX40L has been shown to upregulate SCD1, PMVK and PPARγ, culminating in increased synthesis of monounsaturated fatty acids and cholesterol that are essential for Treg expansion [76]. Similarly, IDO1-kynurenine pathway stimulates expansion of Tregs in a rapamycin-dependent manner [75].

### Treg effector functions in TME

Several metabolic cues have been shown to influence effector Treg functionality. In this regard, hypoxic environments enhance Treg effector function. As demonstrated in a mouse glioma model, HIF-1α stabilization in Tregs leads to a shift toward FAO and glutaminolysis supported by an upregulation of lipid transporters CD36, SLC27A1 and SLC27A4 and decreased glucose oxidation [36]. This increased uptake of lipid is pivotal to suppressive function of Tregs, demonstrated by reduced proliferation of CD8^+^ T cells in HIF-1α KO Tregs [36], whereas treatment with Etomoxir, a mitochondrial inhibitor, depleted CD4^+^Foxp3^+^ T cells population and enhanced antitumor immunity [36]. Genes of pyruvate dehydrogenase kinase 1 (PDK1) and lactate dehydrogenase A (LDHA) were upregulated in Tregs from WT mice but not in HIF-1α KO mice [36]. Recent studies have also shown that elevated glucose metabolism alters Treg functionality. In a study of human tumors, glycolytic genes including Hexokinase 2 (Hk2), glyceraldehyde 3-phosphate dehydrogenase (Gapdh), and Alpha-enolase (Eno1) had higher expression levels in Tregs [76]. In another study, TLR8 signaling selectively inhibits glucose uptake and glycolysis in human Tregs via a downregulation of mTORC1-HIF1a signaling, resulting in reversal of Treg suppression [77]. Indeed, treatment with inhibitors for glucose transporters, glycolysis, cholesterol as well as isoprenoid lipid synthesis have blocked Treg suppressive functions including proliferation of responder T cells and increased number of senescent CD4^+^ T cell population [77]. In addition, adoptive transfer of T cells in a murine model of melanoma enhanced TLR8-dependent tumor regression [77]. However, in B16-F10 melanoma model, deletion of mTORC1 showed reduction in glycolysis and TCA cycle metabolism, while mTORC1-deficient Tregs decreased the expression of suppressive receptors such as CTLA4, ICOS and PD‐1 [78]. Transcriptomic analyses of Tregs isolated from human melanomas demonstrated an increased expression of mitochondrial arginase 2 (ARG2) enzyme and enhanced Treg suppressive capacity [79].

Additionally, several tumor models including mice with ID8 ovarian cancer, MC38 colon cancer, and B16 melanoma have revealed altered Treg biological behavior characterized by substantial Treg apoptosis and potent immunosuppressive phenotype along with efficient inhibition of IL-2 production in effector T cells. Interestingly Treg immunosuppression was not mediated via typical suppressive pathways including CTLA-4, TGF-β, IL-35, or IL-10; instead, it was induced through adenosine production from ATP via CD39 and CD73 signaling [80]. These apoptotic Tregs exhibited an increased susceptibility to reactive oxygen species, which highlights the role of oxidative stress in TME and Treg function [80]. However, selective inhibition of fatty acid-binding protein (FABP5) in ex vivo human natural Tregs triggers mitochondrial DNA release accompanied by disruption in lipid metabolism and oxidative phosphorylation, promoting IL-10-mediated Treg suppressive function [81]. Intriguingly, impairment in electron transport chain (ETC) Complex I and NADH oxidation decreases Treg function [69]. Similarly, loss of mitochondrial complex III lead to decreased expression of genes related to Treg function and suppressive capacity but not proliferation or survival. While RISP (an essential subunit of mitochondrial complex III) KO CD25^+^ Foxp3^+^ mice have shown inhibition of melanoma growth mediated by Treg loss of function under the influence of ten-eleven translocation (TET) family of DNA demethylases [82]. In particular, DNA methylation, 2-hydroxyglutarate (2-HG) and succinate negatively regulated the expression of genes involved in Treg functionality [82].

Other metabolic cues induce an immunosuppressive phenotype in CNS tumors. The constitutive PPAR expression demonstrated increased tumor burden accompanied by an expansion of Treg repertoire in murine models of astrocytoma and oligoastrocytoma [83]. Interestingly, Tregs captured fatty acids threefold higher than CD8^+^ T cells in intracranial murine astrocytoma [84]. In a B16 melanoma model, neuropilin-1 (Nrp1) induced intra-tumoral Treg stability by enhancing quiescence/survival genes, while inhibiting transcriptomic signatures that promote differentiation [85].

## Metabolic pathways involved in heterogeneity of Tregs in the TME

Tregs exhibit distinct tissue-specific heterogeneity in inflammatory conditions and cancer. The reason behind this heterogeneity could be the association of Tregs with tissue-specific transcription factors including PPARγ and GATA3 [86]. Tumor-associated Tregs are often seen at the effector state with distinct metabolic signatures from lymphoid resident Tregs [86]. Cancer cells modify numerous environmental factors and nutrients within the TME, which affects the differentiation and function of tissue-resident Tregs. It has been reported that a sub-population of Tregs in the brain TME express higher level of fatty acid transporters including SLC27A1 and CD36, which favors immunosuppression [36]. However, the attenuation of fatty acid intake inhibits the immunosuppressive characteristics of Tregs in the brain TME [36]. Under physiological conditions, the major energy source of tissue-resident Tregs for their proliferation and function is mediated by mTOR-dependent lipogenesis [87]. Likewise, in the CRC TME, Tregs utilize fatty acid metabolism as a key energy resource to complement glucose metabolism, leading to the accumulation of lipid-intermediates within the TME [76]. It has been reported that OX40^+^ Tregs were accumulated in the visceral adipose tissue (VAT) of obese CRC patients, proposing that VAT might act as a reservoir for OX40^+^Tregs, which subsequently could be migrated to the TME via chemotaxis [88]. A study on plasmacytoid dendritic cells (pDCs) from breast cancer patients showed that excessive accumulation of lactate in the TME enhances tryptophan metabolism and kynurenine secretion by pDCs, leading to the induction of FoxP3^+^ Tregs [89].

It has been reported that Tregs in the CRC TME have Th17 like phenotype, expressing RORγt with elevated IL-17 release [90, 91]. These RORγt^+^FoxP3^+^ Tregs have more stable and immunosuppressive characteristics, compared with RORγt^–^FoxP3^+^ Tregs [91]. Subsequently, studies showed that numerous intermediates from cholesterol metabolism including 24-dehydrocholesterol reductase, 7-dehydrocholesterol reductase act as an agonist for RORγt^+^ in inflammatory conditions [92]. These reports suggest that Th17^+^ effector Tregs found in the CRC TME could be regulated by cholesterol biosynthesis and accumulation of lipid-intermediates. Likewise, highly activated and proliferative Tregs in breast, colorectal, lung and melanoma TME express higher level of CCR8 and is associated with poor survival [51]. Additionally, it has been reported that the tissue-resident Tregs require higher lipid uptake for their function and survival [93].

Accumulating studies demonstrate the cytotoxic properties of circulating and in vitro generated γδ T cells (γδTc), while the functional characteristics of tumor-infiltrating γδTc vary [94, 95]. As mentioned in previous sections, the metabolic microenvironment of the tumor is different with the high supplement of TGF-β1 and IL-10 [4]. This milieu might favor tumor-infiltrating γδTc to acquire certain suppressive characteristics and termed as “γδ T regulatory cells (γδ Tregs)”. It has been reported that two subpopulations of γδTc (Vδ1Tc and Vδ2Tc) have suppressive characteristics with induced FoxP3 expression [96]. However, in renal carcinoma, it has been reported that the expression of FoxP3 was higher in tumor-infiltrating Vδ1Tc, compared with Vδ2Tc [97]. Additionally, the percentage of FoxP3^+^γδTc is inversely correlated with CD8^+^ TILs, confirming the anti-tumor immunosuppressive role for γδTc and poor clinical outcome [98]. Reports suggest that accumulation of γδTc in the TME could be due to the elevated metabolite flux of the mevalonate pathway intermediates [99, 100]. Moreover, isopentenyl diphosphate (IPP), a metabolic intermediate of mevalonate pathway, binds to butyrophilin 3A1 (BTN3A1) and activates γδTc [101]. Moreover, IPP-stimulated Vδ2Tc in the presence of exogenous TGF-β1 and IL-15 could induce the expression of FoxP3 [102]. Altogether, these reports suggest that tumor metabolic milieu could favor the heterogeneity of Tregs, and dampen anti-tumor immune responses.

## Metabolic regulations behind the induction of Tregs in the hypoxic TME

Within the TME, pTregs are generated from naïve CD4^+^ T cells in response to tumor antigens and other stimulatory networks [103]. These induced Tregs have a profound suppressive function alike nTregs [65]. Moreover, studies on tumor antigen-specific Tregs demonstrated that preferential accumulation of pTregs is induced by tumor antigens within the TME could potentially suppress therapeutic vaccinations [103, 104].

Apart from tumor antigens, TME plays an indispensable role to support the generation and accumulation of pTregs. TGF-β, which favors pTregs generation, is involved in the downstream network of HIF-1α and was upregulated in hypoxic microenvironment [105]. The expression of TGF-β and HIF-1α favors each other; HIF-1 promotes the expression of TGF-β in CD4^+^ T cells, and TGF-β attenuates the expression of HIF-1 degrading enzyme prolyl hydroxylase domain 2 (PHD2) and indirectly favors the stability of HIF-1 (Fig. 1b) [105, 106]. A hypoxic microenvironment not only promotes the expression of TGF-β in CD4^+^ T cells, but also their internalization into the cytoplasm. In cytoplasm, TGF-β binds to its receptor and triggers downstream pathways including phosphorylation of SMAD3, binding of phospho-SMAD3 to SMAD4, recruitment of CBP/p300 and binding of these complex on FoxP3 promoter to induce the expression of FoxP3 (Fig. 1b) [107]. Apart from TGF-β, PD-L1 is also a downstream target of HIF-1α. In a hypoxic microenvironment, the expression of PD-L1 on tumor cells was upregulated by HIF-1α and augments their binding with its receptor, PD-1, on T cells [108, 109]. The PD-1/PD-L1 interaction could dephosphorylate PI3K and attenuate the activation of AKT/mTOR pathway, thereby promoting the expression of FoxP3 (Fig. 1b) [108, 109]. Additionally, tumor-derived exosome-mediated release of IL-10 will activate JAK/STAT-3 pathway and stabilizes FoxP3 expression and differentiation of pTregs (Fig. 1b) [110]. Moreover, tumor-derived exosomes not only release IL-10, but also upregulate microRNA-214 (miR-214) [111]. miR-214 enters into T cells through endocytosis and attenuates PTEN pathway and activates PI3K/AKT signaling [111]. These signaling networks activate cycle-associated transcription factor E2F and augment proliferation of nTregs [112]. On the other side, downregulation of PTEN could deplete the expression of CD25 leading to the accumulation of FoxP3^+^CD25^–^ pTregs [113].

It has been reported that TGF-β-induced Tregs express reduced level of GLUT-1 and have lower glycolysis and higher oxidative phosphorylation in the TME [76]. Moreover, the reduction in glycolysis could dampen mTOR signaling and favor the generation of pTregs. The switch between glycolysis to oxidative phosphorylation is considered as a key metabolic checkpoint for pTreg generation in the TME [114]. In the TME, HIF-1α-dependent transcriptional network facilitates glucose metabolism, which determines the polarization of T cell choice between pTregs and Th17; Th17 polarization requires higher glycolysis but not for pTregs [115]. Likewise, acetyl-coA carboxylase 1 (ACC1), a major enzyme in fatty acid anabolism, also determines the fate of Th17 and Treg polarization. Inhibition of ACC1 favors pTregs, while activation favors Th17 differentiation [116, 117]. Unlike pTregs, nTregs persist in distinct tumor metabolic environment. Highly proliferative and immunosuppressive nTregs require mTOR activation and higher glycolysis and fatty acid metabolism for their survival [118, 119]. In the tumor milieu, glucose-deprived condition favors more of pTregs than nTregs in order to balance the percentage of Tregs within the TME [103].

## Factors affecting the Treg metabolism

### Vitamins

Vitamins, including A, B, C and D, play important regulatory roles, which affect different metabolic pathways, modulate gene transcription and immunological responses [120]. Vitamin A metabolite, retinoic acid (RA), produced by specific subsets of dendritic cells (DCs) can regulate FoxP3 expression via direct or indirect means [121–123]. RA can directly promote FoxP3 expression by increasing histone methylation and acetylation of the conserved non-coding DNA sequence (CNS) at the FoxP3 gene locus and promoter region, and by triggering the activation of extracellular-related kinase (ERK) signaling [122]. Through indirect means, RA supports the stability of FoxP3 expression [121] and promotes Treg survival and expansion via the activation of IL-2 signaling, leading to the conversion of TGF-β-mediated CD4^+^CD25^+^FoxP3^+^ Tregs from CD4^+^CD25^–^FoxP3^–^ T cells [124, 125].

Other vitamins such as C and D have also been implicated in the modulation of FoxP3 expression. Studies on induced Tregs (iTregs) demonstrated the importance of vitamin C in stabilizing FoxP3 expression via the induction of the TET-mediated demethylation of CNS2 region, which in turn activates FoxP3 gene transcription [126, 127]. TET family proteins are enzymes, which facilitate DNA demethylation leading to the activation of gene transcription [128]. Studies have shown that deletion of both TET2 and TET3 genes is sufficient to disrupt the stability of FoxP3 expression and that deletion of TET2 gene in Tregs counteracts the effect of vitamin C and diminishes the suppressive activity of Tregs [126, 127, 129]. In an allogeneic skin transplantation model, vitamin C-treated allogenic iTregs exhibited a high suppressive property with increased expression of Treg gene signature and a stable expression of FoxP3 induced by the TET-mediated demethylation [130].

Vitamin D3 metabolites, 25-dihydroxyvitamin D3 [25(OH)VD3] and the active form 1,25(OH)2VD3 are also known to induce the expression of FoxP3 in CD4^+^ T cells upon the stimulation of TCR and IL-2 signaling [131, 132]. Jeffery et al. demonstrated that stimulating CD4^+^CD25^–^ T cells with 1,25(OH)2VD3 diminished the production of proinflammatory cytokines (IFNγ, IL-17 and IL-21), and in cooperation with IL-2 significantly upregulated the levels of CTLA-4 and FoxP3 [131]. Indeed, vitamin D response element (VDRE) has been found in the intronic CNS region of the human FoxP3 gene, suggesting that this region could serve as a functional enhancer for the induction of FoxP3 gene expression [133]. However, the exact mechanisms by which VDRE transcriptionally regulates FoxP3 gene remain undetermined. Data from clinical trials supported the efficacy of vitamin D in promoting the expansion of peripheral Tregs in patients with inflammatory or autoimmune conditions [134], suggesting that targeting vitamin D in Tregs could offer a therapeutic efficacy in cancer patients.

Vitamin B3 is another vitamin which is known to regulate the generation of Tregs present in the colon and to maintain colonic immune tolerance [135, 136]. Additionally, niacin, a form of Vitamin B3, triggers anti-inflammatory signals through G protein-coupled receptor (GPR) 109a, leading to expression of RA synthetases in colon macrophages and DCs, which in turn stimulate Treg differentiation [135].

### Metabolites

Metabolites generated from amino acid catabolism can positively influence Treg induction and function. The expression of IDO, an enzyme which catabolizes the amino acid tryptophan and limits its availability to T cells, showed a positive correlation with Treg density in tumors, for instance in papillary thyroid carcinomas [137]. DCs expressing IDO can induce the generation of FoxP3^+^ Tregs from naïve T cells, and block the differentiation of Th17 cells [138–140]. IDO catabolizes tryptophan to kynurenine, which in turn binds to AHR and facilitates the differentiation of naïve T cells to Tregs [141, 142]. Moreover, the ability of the tryptophan metabolite, 3-hydroxyanthranilic acid (3-HAA), in increasing the expression of TGF-β in DCs and levels of Tregs and in reducing the levels of Th1 and Th17 cells has been demonstrated in vivo. Alternatively, limited availability of tryptophan mediated by IDO activity can trigger a stress response pathway via the activation of general control nonderepressing-2 (GCN2) protein kinase, resulting in the inhibition of mTORC2 and Akt activation, thereby favoring Treg differentiation/function/stability and inducing cell cycle arrest and anergy in T effector cells [40, 143, 144]. Collectively, these data implicate that tryptophan catabolism could be essential for the maintenance of Treg stability, and blocking this pathway could offer a therapeutic benefit for targeting Treg metabolism.

Metabolites from purine (nucleotide base) catabolism can regulate Treg induction and FoxP3 expression by the generation of extracellular adenosine triphosphate (ATP), which binds to the purinergic P2X7 receptor, and leads to the disruption of FoxP3 stability and promotion of Treg conversion to Th17 cells [145]. However, excess extracellular ATP can be converted by CD39^+^CD73^+^ Tregs into immunosuppressive adenosine [146, 147]. Adenosine in turn acts on effector T cells via the binding to its receptor (A2AR) and exert suppressive functions, including the inhibition of TCR signaling and the induction of cell cycle arrest [148, 149]. Thus, targeting metabolic pathways resulting in excess production of ATP could be important to control the adenosine-mediated immunosuppression pathway.

## Targeting Treg metabolism or metabolic pathways

Conventional cancer therapeutic strategies such as chemotherapy and radiotherapy can effectively reduce the number of activated Tregs and increase the number of effector T cells in cancer patients [150–152]. For instance, Cao et al. reported that gamma irradiation can reduce the suppressive function of Tregs by downregulating the expression of FoxP3 and membrane TGF-β [153]. Moreover, clinical trials showed the efficacy of chemotherapy in reducing the frequency of CD4^+^CD25^+^FoxP3^+^ Tregs and their suppressive function in the circulation of patients with hepatocellular carcinoma [154] and in tumor tissues of breast cancer patients [155]. However, there are multiple potential risks associated with cytotoxicity, which can arise from using chemotherapeutic and radiotherapeutic drugs as these strategies could also affect the number of effector T cells and negatively modulate anti-tumor immunity [152, 156]. Additionally, evidence from animal models suggests that emergence of Treg resistance against radiotherapy is mediated by the overexpression of glucocorticoid-induced tumor necrosis factor receptor family-related protein (GITR) and increased production of TGF-β [153, 157]. In addition, a study by Muroyama et al. showed that radiotherapy in tumor-bearing mice increased the suppressive function and proliferation of tumor-infiltrating Tregs and the expression of inhibitory immune checkpoints, such as CTLA-4, on Tregs [158]. Schuler et al. reported increased frequency of circulating Tregs and Treg suppressive function in patients with head and neck squamous cell carcinoma (HNCC) following chemo-radiotherapy [159], while Oweida et al. showed that tumor-infiltrating Tregs can induce resistance against radiotherapy in mouse HNCC model and their blockade using anti-CD25 mAb in combination with radiotherapy showed better clinical outcomes [160]. Therefore, these latter findings indicate that identifying Treg-targeting agents is crucial to maximize anti-tumor efficacy and specifically deplete Tregs with minimal or no adverse effects on effector T cells.

Effects of chemotherapy and radiotherapy on Treg metabolic reprograming have not been yet reported. Nonetheless, Treg metabolism could be targeted via the inhibition of various metabolism-related signaling mediators, such as PTEN, HIF-1α, TGF-β and AMPK, in addition to key enzymes which facilitate fatty acid metabolism (such as ACC1) or amino acid catabolism (such as IDO) as described below.

**Fig. 2:**
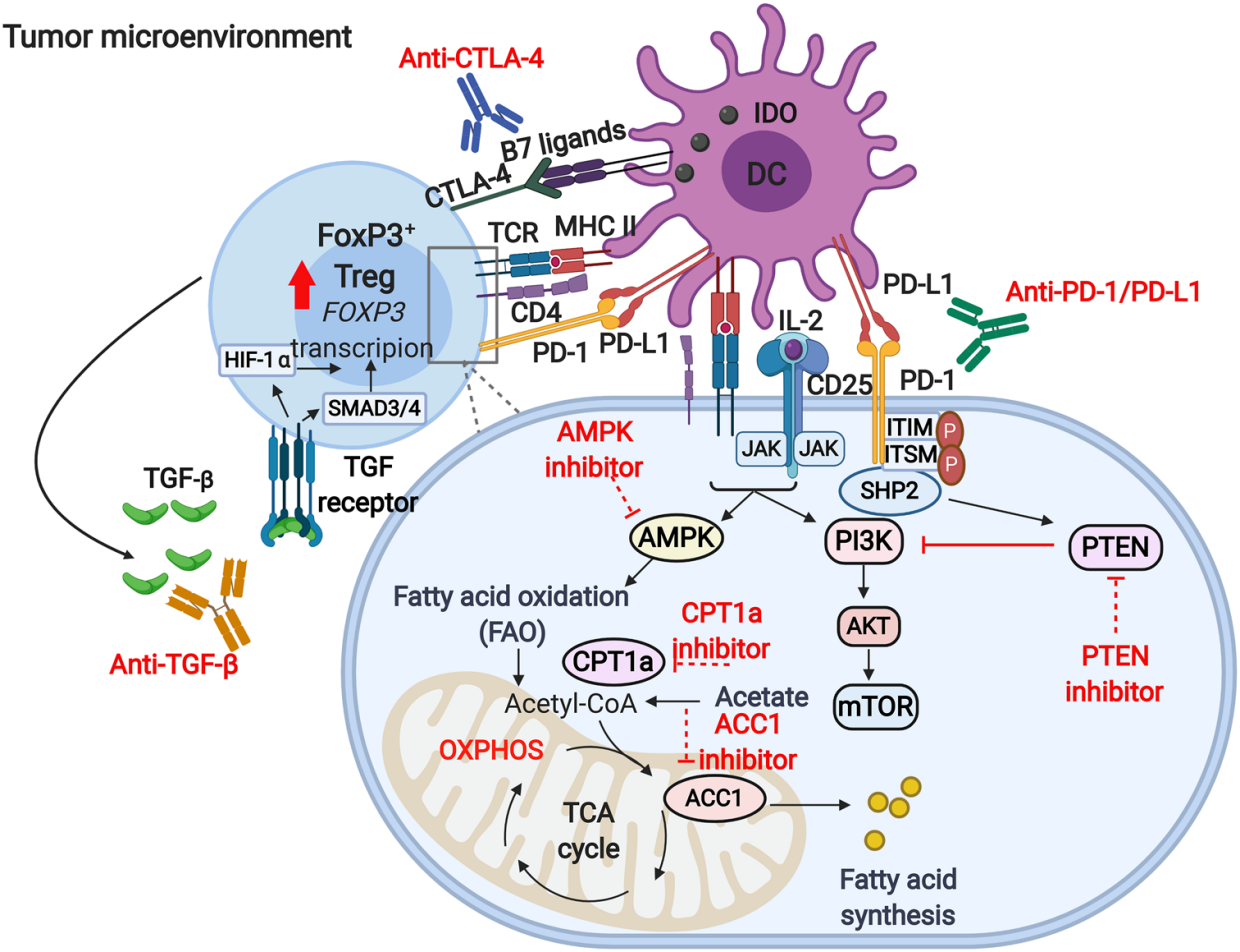
Strategies for targeting Treg metabolism. The accumulation of high numbers of Tregs within the tumor microenvironment could be governed by the action of various mediators and signaling pathways. Direct interactions between tolerogenic DCs and Tregs results in the activation of TCR-mediated signaling pathway. Moreover, the interactions between CTLA-4 on Tregs and B7 ligands on DCs induces IDO expression, an enzyme which facilitates tryptophan catabolism and limits its availability to T effector cells. Metabolites from tryptophan catabolism can be also important for Treg induction and FoxP3 stability (not shown here). IL-2/CD25 signaling is essential for the survival and proliferation of Tregs, as it leads to the downstream activation of PI3K/AKT/mTOR signaling and STAT5 signaling. PD-1/PD-L1 signaling, on the other hand, suppresses the activation of PI3K/AKT/mTOR via PTEN, thereby favoring FoxP3 expression and stability. TCR-mediated signaling in cooperation with IL-2 signaling triggers the activation of AMPK, a critical protein kinase for lipid metabolism and FAO, which are required for energy production, and Treg survival and function. ACC1 is an enzyme which facilitates fatty acid synthesis, while CPT1a is responsible for FAO. Activated, highly immunosuppressive Tregs can release high levels of TGF-β, which is a key mediator for Treg survival, function and differentiation. Via an autocrine signaling, the activation of TGF-β signaling in Tregs can induce HIF-1α expression and trigger the activation of SMAD3 and 4 signaling, which subsequently prompt FoxP3 expression and Treg function. Targeting PD-1 and CTLA-4 signaling by mAbs could be beneficial in reducing FoxP3 stability and diminishing Treg numbers. Small molecule inhibitors targeting PTEN, ACC1 and CPT1a could offer a therapeutic benefit in cancer by destabilizing FoxP3 expression and suppressing Treg function. Moreover, the neutralization of TGF-β could block the HIF-1 and SMAD-mediated FoxP3 induction, and the inhibition of AMPK activity could be beneficial in depleting Tregs and disrupting FoxP3 expression. Potential therapeutic inhibition strategies are indicated by dotted red lines

PI3K/AKT/mTOR signaling pathway is amongst the key pathways, which controls metabolic reprogramming in Tregs and negatively regulates Treg suppressive function. Conversely, the inhibition of PI3K/AKT/mTOR pathway promotes the programming of immunosuppressive Tregs [161, 162]. Studies have shown that inhibiting the activity of specific isoforms of PI3K or mTORC1 (target of rapamycin complex 1) can diminish the expression of inhibitory immune checkpoints such as PD-1 and CTLA-4 and negatively influence Treg phenotype [78, 163]. A study by Kanamori et al. reported that FoxP3^+^ Tregs can be generated upon the reprogramming of Th1 cells via therapeutic intervention targeting the activation of PI3K/AKT/mTOR pathway, causing a metabolic shift from glycolysis to OXPHOS [164]. Another study by Basu et al. showed that Treg stability can be disrupted by the pharmacological activation of AKT, which in turn leads to an increase in glucose uptake and glycolysis [165]. The stability of Tregs and their suppressive phenotype requires PTEN (phosphatase and tensin homolog), a key negative regulator of PI3K/AKT signaling, which restrains the capacity of Th1 and follicular T helper cell polarization [113, 166]. Studies have shown that FoxP3 instability in PTEN-deficient Tregs is more likely to occur as a result of increased glycolysis and reduced OXPHOS [113, 144, 166]. A study in melanoma mouse model showed that PTEN is required for the IDO-induced Treg activation and stability [144]. It was shown that IDO inhibits the phosphorylation of serine residue 473 on AKT and mTOR/TORC2 complex and hence hampers their activation [144]. On the other hand, in vivo administration of IDO inhibitor in tumor-bearing mice resulted in the phosphorylation of Akt in Tregs [144]. PD-1 expressed by activated Tregs is another inhibitor of AKT through the activation of PTEN and therefore has been implicated in Treg stability [167, 168]. Altogether, these findings suggest that targeting PTEN, IDO and PD-1 signaling pathways in Tregs can lead to metabolic programming switching from lipid oxidation to glycolysis via the activation of PI3K/AKT/mTOR signaling in activated Tregs (Fig. 2). Since CTLA-4 is constitutively expressed by Tregs and may play a key role in stabilizing FoxP3 expression via the induction of IDO expression [56, 57, 149]; hence, targeting CTLA-4 could be beneficial in altering Treg programing and disrupting Treg stability (Fig. 2).

T cell metabolism is a highly dynamic process and can be mimicked in vitro using experimental conditions, which include the optimization of TCR signal strength (dose and duration) and the presence of cytokines or drugs [169, 170]. The metabolic requirements for Treg differentiation in vitro under the influence of cytokines have been well studied; however, less is known about the metabolic factors required for Treg induction in vivo. Highly activated immunosuppressive Tregs rely on fatty acid metabolism, rather than glycolysis; hence, targeting enzymes which facilitate fatty acid metabolism, such as ACC1, could offer a therapeutic benefit in cancer by depleting activated Tregs. The requirement of lipid uptake and oxidation for FoxP3 expression in T cells has been determined through the use of etomoxir, a small molecule inhibitor, which targets fatty acid oxidation (FAO) selectively by inhibiting the activity of carnitine palmitoyltransferase 1a (CPT1a), resulting in the abrogation of FoxP3 expression [37, 171]. Moreover, pharmacological inhibition of enzymes regulating the generation or signaling of fatty acid derivatives such as estrogen-related receptor-α (ERRα) can also impair Treg differentiation and function in vitro [172]. On the contrary, the differentiation of Tregs can be rescued upon the addition of fatty acids to in vitro cultures resulting in the upregulation of ERRα and lipid oxidation [172]. These data implicate that pharmacologic inhibitors targeting FAO could be potentially used to block Treg programing and disrupt their stability by affecting FoxP3 expression (Fig. 2).

Hypoxia and HIF1‐α can be also involved in the induction of FoxP3 expression [24, 25, 105]. However, some studies have demonstrated that HIF1‐α expression can impair Treg stability in vivo as it induces the transcription of glycolytic genes and promotes FoxP3 degradation [115, 173, 174]. Suppression of HIF1‐α function via the activation of oxygen‐sensing prolyl‐hydroxylase (PHD) results in the induction of Treg programming in metastatic niches [175]. Alternatively, targeted deletion of the HIF1‐α E3 ubiquitin ligase in Tregs can increase the expression of HIF1‐α, which can directly bind to the promoter of the IFNG gene and trigger the expression of IFN‐γ in Tregs, resulting in their conversion into Th1‐like cells [176, 177]. Considering the mutual relationship between TGF-β and HIF-1α, inhibition of TGF-β signaling could result in effective depletion of Tregs by limiting the capacity of FoxP3^+^ Treg survival and differentiation [147] and increase the degradation of HIF-1α (Fig. 2).

AMP-activated protein kinase (AMPK) signaling is another pathway, which can promote the generation of Tregs and diminish the numbers of Th1 and Th17 cells [37], thereby favoring immunosuppression and tumor progression. LKB1, upstream of AMPK, is another metabolic sensor, which is critical for lipid metabolism, OXPHOS, energy production and survival and function of Tregs in an independent manner of the AMPK pathway [178]. Collectively, these studies suggest that targeting AMPK or LKB1 signaling may be beneficial in regulating Treg metabolisms and disruption of Treg stability. The potential metabolic pathways, which could be targeted for the improvement of disease prognosis, are depicted in Fig. 2.

## Concluding remarks and future perspectives

The physicochemical properties of the TME could be altered by nutrient availability and metabolic reprogramming of tissue-resident cells. These modifications are evident by the development of hypoxic environment and reduction in pH, due to changes in cell-mediated transcription factors and the accumulation of metabolic intermediates [179]. Besides the biochemical cues, which have been explained throughout the above sections, physical modifications could also be greatly influenced by the metabolic alterations within the TME [180]. These physical cues could alter the cellular characteristics, including proliferation, metastatic potential and stem cell features of tumor, as well as other tissue-resident cells [180]. The major physical cues affect the extra cellular matrix (ECM) of the TME, including their pore size, alignment of fibrous tissue, cellular attachment and cross-link of collagen, which favor tumor progression [181]. Indeed, it has been reported that an intact ECM component, high molecular weight hyaluronan (HMW-HA), could stabilize FoxP3 and favor the survival and differentiation of Tregs with in the ECM [182]. Additionally, HWA-HA could enhance the suppressive function of Tregs in both in vivo and in vitro [182].

In all cases, the metabolic modifications in the TME favor tumor progression and tumor-immune evasion. In addition, immunometabolism has also been manipulated to enhance current immunotherapeutic modalities through adoptive T cell transfer. For instance, the metabolic potential of genetically-engineered Chimeric Antigen Receptor (CAR)-T cells or other T cells, including T effector cells, could be enhanced to overcome the deleterious effects of the TME and enhance their anti-tumor aptitude [183, 184]. Moreover, the plausible side-effects of metabolic modulators on non-malignant tissue have not been fully elucidated. However, reports showed that the deleterious effects of metabolic inhibitors are very low, compared with other drugs, perhaps due to the high metabolic plasticity of tissue-resident cells [183, 185].

This review mainly resolves three important queries (1) how does the hypoxic metabolic TME affects the function of Tregs, (2) how do pTregs benefit from the metabolic cues within the TME and (3) what possible therapeutic modalities can be employed to target the metabolic reprogramming of Tregs. Infiltration of Tregs is considered as a hallmark of the TME and can affect the progression and metastasis of tumor. The metabolic reprogramming within the TME influences Tregs in three main ways; (1) encouraging the trafficking of Tregs to the TME, (2) induction of Tregs from conventional T cells and (3) upregulating the immunosuppressive characteristics of Tregs. The major metabolic pathways which could influence the Tregs in the TME include tryptophan metabolism, glycolysis and fatty acid oxidation. Moreover, the metabolic reprogramming of Tregs is not limited to a certain pathway. For instance, glycolysis could affect Tregs in conflicting ways; favoring the proliferation of Tregs and attenuating their suppressive function [186]. However, the intermediate metabolites of glycolysis and tryptophan metabolism promote the regulatory function of Tregs [186].

Several factors which are indispensable for the effective function of effector T cells are restricted in the TME, while metabolic alterations favor unrestrained tumor proliferation that leads to nutrient-deprived milieu, excess accumulation of metabolic intermediates, and inadequate oxygen supply. These conditions benefit the generation of pTregs through HIF-1-dependent manner. Moreover, the reduction in glycolysis triggers AMPK-mediated mTOR inhibition, which is the foremost signaling pathway for the generation of pTregs in the hypoxic tumor milieu.

Next, we focused on the development of therapeutic modalities, which could work synergistically with current immunotherapies for better prognostic outcomes. Up to date, there is evidence demonstrating that utilizing mAbs against immune checkpoints, suppressive mediators (e.g., TGF-β), epigenetic modifiers and pharmacological inhibitors for protein kinases, fatty acid transporters, fatty acid metabolism-related enzymes which favor FAO pathways, Treg differentiation and suppressive functions could have a promising clinical impact in cancer patients. However, more studies utilizing advanced technologies are required to investigate the epigenetic and molecular signaling driven by metabolic reprogramming, which influence the differentiation of Tregs and their suppressive and migratory properties. For instance, single cell RNA sequencing could be adapted to uncover the molecular pathways involved in Treg metabolic reprogramming during cancer progression. Additionally, the utilization of Assay for Transposase-Accessible Chromatin sequencing (ATAC-seq) could be used to analyze the transcription factors which have key roles in regulating epigenetic mechanisms and mediate metabolic alterations shaping Treg stability, plasticity and function. Furthermore, in situ imaging and metabolic profiling could be utilized to analyze cell-to-cell communication between Tregs and other cell types to reveal novel metabolic interactions, which potentially could provide new Treg metabolic interventions for cancer treatment.
